# 988 Comparative Effectiveness of Interventions for Post-Burn Neck Contractures: A Systematic Review and Meta-Analysis

**DOI:** 10.1093/jbcr/iraf019.519

**Published:** 2025-04-01

**Authors:** Divya Hegde, Dhaval Bhavsar

**Affiliations:** The University of Kansas Health System Burnett Burn Center; The University of Kansas Health System Burnett Burn Center

## Abstract

**Introduction:**

Post-burn neck contractures, characterized by restricted movement due to scar tissue formation, significantly impact both function and aesthetics. Numerous treatment options exist, including surgical interventions, physical therapy, and assistive devices. We wanted to perform systematic review of different interventions for post burn neck contracture release.

**Methods:**

We conducted a systematic search across databases including PubMed, CINAHL, and Cochrane from January 2024 to March 2024. The inclusion criteria encompassed randomized controlled trials (RCTs), observational studies, and case series examining interventions for post-burn neck contracture. Outcome measures included range of motion (ROM), pain scores (Visual Analog Scale), contracture recurrence, graft take, and complication rates. Data was analyzed following Cochrane protocols and PRISMA guidelines.

**Results:**

A total of 24 studies were included, comprising of both surgical and non-surgical interventions. Surgical methods, particularly the use of free flaps, demonstrated the most significant improvement in ROM and patient mobility, with lower rates of contracture recurrence compared to skin grafts and tissue expansion techniques. Non-surgical interventions, while less effective in severe cases, showed moderate improvements in ROM and pain reduction. However, the lack of standardized outcome measures across studies presented challenges in direct comparison.

**Conclusions:**

The variability in study designs and outcome measures highlights the need for standardized metrics in future research to better assess and compare treatment effectiveness.

**Applicability of Research to Practice:**

Future studies should aim to standardize outcome measures to facilitate more straightforward comparisons and meta-analyses. Incorporating a core set of outcomes, including pain levels (VAS), graft take percentage, complication rates, and range of motion, would provide a balanced view of both the patient’s subjective experience and objective clinical success. Additionally, longitudinal studies with extended follow-up periods would be beneficial to assess the durability of these outcomes. For example, evaluating the range of motion and complication rates at multiple time points post-surgery could provide insights into the long-term success and potential late-onset issues of the treatments.

**Funding for the Study:**

N/A

